# *Wuchereria bancrofti* infection is associated with progression to clinical visceral leishmaniasis in VL- endemic areas in Muzaffarpur, Bihar, India

**DOI:** 10.1371/journal.pntd.0011729

**Published:** 2023-10-30

**Authors:** Abhishek Kumar Singh, Tanyth de Gooyer, Om Prakash Singh, Sundaram Pandey, Aziza Neyaz, Kristien Cloots, Sangeeta Kansal, Paritosh Malaviya, Madhukar Rai, Susanne Nylén, Jaya Chakravarty, Epco Hasker, Shyam Sundar

**Affiliations:** 1 Infectious Disease Research Laboratory, Department of Medicine, Institute of Medical Sciences, Banaras Hindu University, Varanasi, India; 2 Department of Public Health, Institute of Tropical Medicine, Antwerp, Belgium; 3 Department of Biochemistry, Institute of Sciences, Banaras Hindu University, Varanasi, India; 4 Department of Community Medicine, Institute of Medical Sciences, Banaras Hindu University, Varanasi, India; 5 Department of Microbiology, Tumor and Cell Biology, Karolinska Institutet, Solna, Sweden; Erasmus MC, University Medical Center Rotterdam, NETHERLANDS

## Abstract

**Background:**

Co-endemicity of neglected tropical diseases (NTDs) necessitates that these diseases should be considered concomitantly to understand the relationship between pathology and to support disease management and control programs. The aims of the study were to assess the prevalence of filarial infection in asymptomatic *Leishmania donovani* infected individuals and the correlation of *Wuchereria bancrofti* infection with progression to clinical visceral leishmaniasis (VL) in Bihar, India.

**Methodology/Principal findings:**

Within the Muzaffarpur-TMRC Health and Demographic Surveillance System (HDSS) area, a cohort of *Leishmania* seropositive (n = 476) or seronegative individuals (n = 1130) were sampled annually for three years for filarial infection and followed for progression to clinical VL. To corroborate the results from the cohort study, we also used a retrospective case-control study of 36 VL cases and 71 controls selected from a subset of the HDSS population to investigate the relationship between progression to clinical VL and the prevalence of filarial infection at baseline. Our findings suggest a higher probability of progression to clinical VL in individuals with a history of filarial infection: in both the cohort and case-control studies, progression to clinical VL was higher among filaria infected individuals (RR = 2.57, p = 0.056, and OR = 2.52, p = 0.046 respectively).

**Conclusion:**

This study describes that progression to clinical VL disease is associated with serological evidence of prior infection with *W*. *bancrofti*. The integration of disease programs for *Leishmania* and lymphatic filariasis extend beyond the relationship of sequential or co-infection with disease burden. To ensure elimination targets can be reached and sustained, we suggest areas of co-endemicity would benefit from overlapping vector control activities, health system networks and surveillance infrastructure.

## Author summary

India has committed to eliminate two neglected tropical diseases (NTDs) as a public health problem: visceral leishmaniasis (VL) and lymphatic filariasis (LF), both of which are transmitted by vectors and are caused by the parasites *Leishmania donovani* and *Wuchereria bancrofti*, respectively. Notably, the majority of *L*. *donovani* infections do not lead to a clinical event of VL and asymptomatic infections significantly outnumber the clinical cases. While mixed infections with *Leishmania* parasites and those causing lymphatic filariasis have been reported, the influence of the immune response triggered by filarial infection on the clinical manifestations and progression of both disorders is not well understood.

The Muzaffarpur-TMRC Health and Demographic Surveillance System (HDSS) in Bihar is a well-established demographic platform in an area with the highest incidence of *L*. *donovani* infections in India which is also co-endemic for lymphatic filariasis. We used both cohort and case-control study designs within the HDSS population to assess the prevalence of filarial infection in blood samples and examine the association between filarial infection and the progression to clinical VL.

Our findings strongly suggest there is an increased risk of progression to clinical VL in individuals with a history of infection with *W*. *bancrofti*: in both the study designs, progression to clinical VL was higher among filaria-infected individuals. These results highlight that the co-endemicity of these NTDs necessitates an integrated approach in diagnosis and disease management to ensure elimination targets can be achieved and sustained.

## Introduction

Neglected tropical diseases (NTDs) typically affect the poorest populations, and the co-endemicity of several NTDs necessitates an integrated approach in diagnosis and disease management to ensure elimination targets can be achieved and sustained. Specifically, India has committed to eliminate two NTDs as a public health problem: visceral leishmaniasis (VL) and lymphatic filariasis (LF) [1]. By June 2022, the VL elimination program in India had been successful in reducing the incidence of VL below the elimination target of less than one case per 10,000 population annually in 99% of sub-districts (blocks) [2]. Progress towards LF elimination in India has been slower: a total of 257 LF endemic districts exist in the country [3], with the mass drug administration (MDA) programs facing several challenges including poor community participation and low compliance [4].

Leishmaniases are protozoan diseases caused by different *Leishmania* spp: all are transmitted by the bite of infected female phlebotomine sandflies, and all are intracellular parasites of macrophages in their mammalian host. Visceral leishmaniasis or kala-azar is a systemic disease and is the most severe form of *Leishmania* infection. VL affects bone marrow, spleen, liver and lymph nodes and is usually fatal if left untreated. In India, VL is caused by *Leishmania donovani* and has an anthroponotic transmission pathway (human–sandfly–human). Most *L*. *donovani* infections do not lead to a clinical event of VL and those asymptomatic infections significantly outnumber the clinical cases [5,6]. The role of asymptomatic infections in maintaining disease transmission is not well understood [7,8], however mathematical modelling suggests that these individuals may act as a parasite reservoir and remain infective to the sandfly vector [9]. We have previously described a correlation between high baseline antibody levels (titres) and progression to clinical VL in cohorts from India and Nepal [10,11]. The outcome of infection with *L*. *donovani* depends on the host immune response [12,13], which can be influenced by other factors, such as co-infection with other pathogens, evident in the case of HIV/AIDS. The impact of infections such as helminths, that induce and are being controlled by immune responses opposing those needed to control intracellular pathogens of macrophages, can also be envisaged to interfere with *Leishmania*. Parasitic worms typically drive and are controlled by Type 2 immune responses [14], while *Leishmania* and other intracellular pathogens of macrophages (e.g. mycobacteria) depend on Type 1 responses for control [13]. In addition to driving Type 2 immune response, many chronic worm infections, including filarial infections, are also strong drivers of regulatory responses, needed to limit infection-induced tissue pathology [15–19]. These regulatory responses dampen immunity more broadly and may favour leishmanial replication [20,21]. A study in Brazil found that a higher proportion of patients who were co-infected with helminths had persistent cutaneous leishmaniasis (CL) lesions due to *Leishmania braziliensis* at the end of the 90-day study period compared to those who were helminth-negative [22]. While mixed infections with *Leishmania* parasites and those causing lymphatic filariasis have been reported in areas of co-endemicity [23,24], the influence of the immunological modulation triggered by filarial infection on the clinical manifestations and progression of both disorders is not well understood. A recent study from South India suggests that underling filarial infection can impact cytokine patterns to favour bacterial replication in tuberculous lymphadenitis [25].

Lymphatic filariasis (LF) is a mosquito-borne parasitic disease predominantly caused by infection with the nematode parasite *Wuchereria bancrofti* (more than 90% of cases), but also *Brugia malayi* and *B*. *timori* [26]. LF parasites have a complex life cycle: third-stage filarial larvae will enter the skin following a bite wound from an infected mosquito and migrate to the lymphatic vessels where they develop to an adult stage. Adult filarial worms can live for several years in the lymph nodes and vessels. Female worms release first-stage larvae called microfilaria which circulate periodically in the host’s blood system and are taken up by mosquitos which feed on an infected host. Acute or chronic forms of filarial infection can result in severe complications of lymphedema (elephantiasis) and hydrocele, causing disfigurement and physical disability. However, the majority of filarial infections are asymptomatic—although these still affect the lymphatic, renal and immune systems, despite showing no outward signs of disease. Asymptomatic individuals remain infectious, and reducing the density of parasites circulating in the blood of infected persons through mass drug admission (MDA) to all people living in LF endemic areas is an important control measure to interrupt transmission. LF infection can be diagnosed by various methods, including the identification of microfilariae in nocturnal blood smears, using PCR assays targeting the repeat regions of *W*. *bancrofti*, detecting circulating filarial antigen (CFA) in the blood (a surrogate for viable adult worms), or by detecting anti-filarial antibodies using serological immunoassays [27].

In India, Bihar has the highest incidence of anthroponotic *Leishmania donovani* infections [28] with 547 cases were reported in 2022, although this has declined from previous years [29]. In 2021, the highest burden of lymphoedema cases (89,970; 6.98 per 10,000 population) were reported from Bihar [30] where all 38 districts are endemic for lymphatic filariasis (caused by *Wuchereria bancrofti*) [3]. While India began MDA with anti-filarial drugs in 1997, coverage and compliance has been irregular [31]. Consistent data on the coverage of MDA specifically in Bihar state is not readily available. The Muzaffarpur-TMRC Health and Demographic Surveillance System (HDSS) was established in 2007 with funding by the National Institutes of Health (NIH, USA) under its Tropical Medicine Research Centres (TMRC) grants and covers an area of 66 villages with a population size of >125,000 in the Muzaffarpur district in Bihar state (described in [32]). The majority of these villages (50/66) are located in a geographically contiguous area of 68 km^2^ in the Kanti and Marwan blocks of Muzaffarpur, while the remaining 16 villages are located in seven other blocks [32]. The Muzaffarpur-TMRC HDSS has been valuable in examining factors that influence the prevalence, distribution and pathogenesis of VL and other neglected tropical diseases prevalent in rural Bihar, and for monitoring trends in transmission in an elimination setting [10,11,33,34]. The HDSS provides a unique opportunity to examine the association between LF and progression to VL disease, due to the intensified routine follow up of the population with regard to health parameters including VL.

During the sixth demographic survey in 2013 which covered a Muzaffarpur TMRC-HDSS population of 105,874 we documented a possible association between LF and VL (described in our approved study protocol DMID protocol number: 17–0053; 30 November 2019). When household members were asked about the presence of signs of LF (lymphedema, elephantiasis or hydrocele), the LF prevalence rate was 5.1% in the overall HDSS population but 10.4% among those who had suffered from VL since January 2007 (RR: 2.1, 95% CI: 1.5–3.0). The present study aimed to assess the prevalence of filarial infection in blood samples from asymptomatic *L*. *donovani* infected individuals and examine the association between filarial infection and the progression to clinical VL.

## Materials and methods

### Ethical approval

This study is a component of Muzaffarpur NIH-TMRC HDSS for which ethical approval was received from the Institutional Review Boards of the Institute of Medical Sciences, Banaras Hindu University (BHU), Varanasi, India (Dean/2017/EC/185 dated: 24.10.2017), and the review committee of the U.S. National Institutes of Health-National Institute of Allergy and Infectious Diseases (NIH-NIAID) (reviewed and accepted as part of the award process through grant No.: 5U19AI074321). The Institutional Review Board (IRB) of BHU has received accreditation from the National Institutes of Health in the United States. The study was conducted in accordance with the Declaration of Helsinki. Informed consent was obtained in writing from all participants involved in the study. In the event of illiterate participants, a thumb print and the signature of a neutral third party were used alternatively. The consent of a parent or legal guardian was obtained from any participant who was under the age of 18. All data was anonymized.

### HDSS population

**Fig 1:**
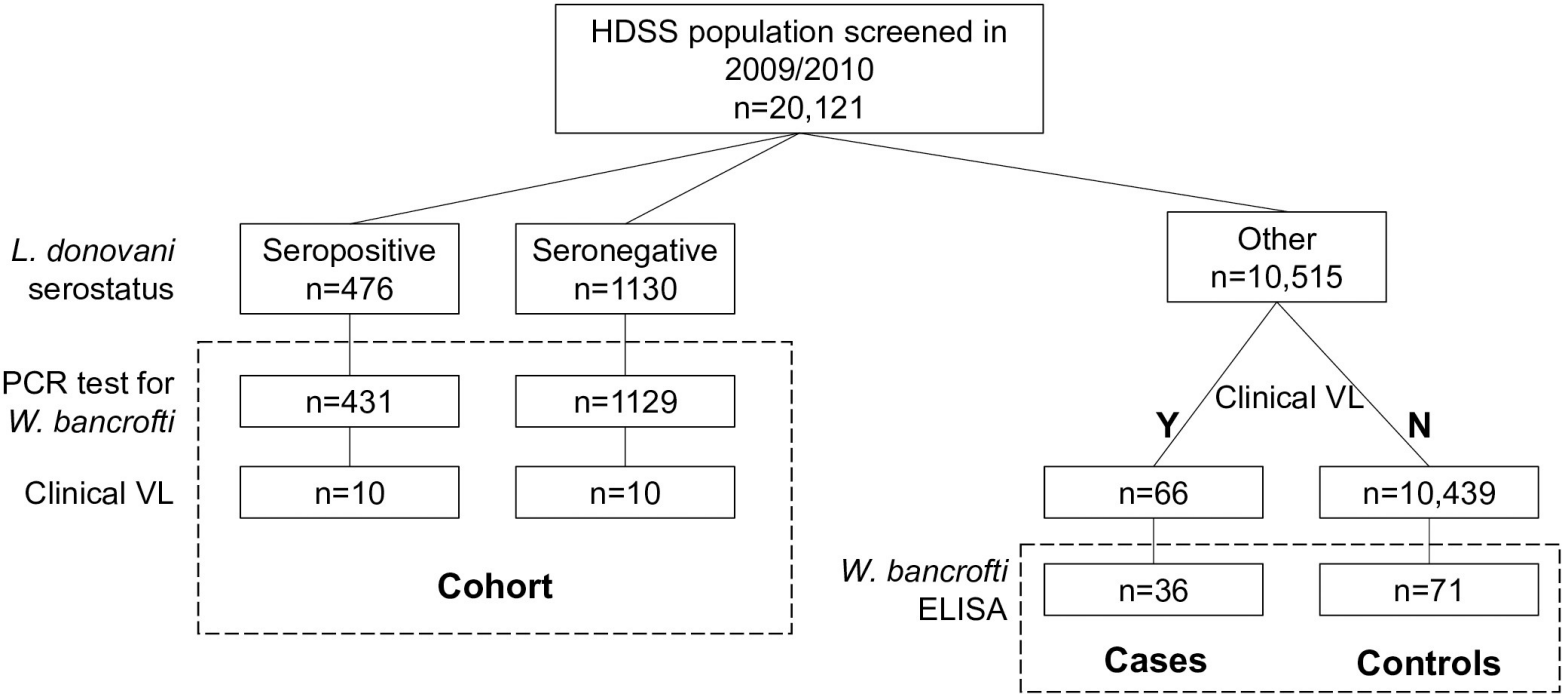
Selection of sample for cohort and case-control study from HDSS population.

**Fig 2:**
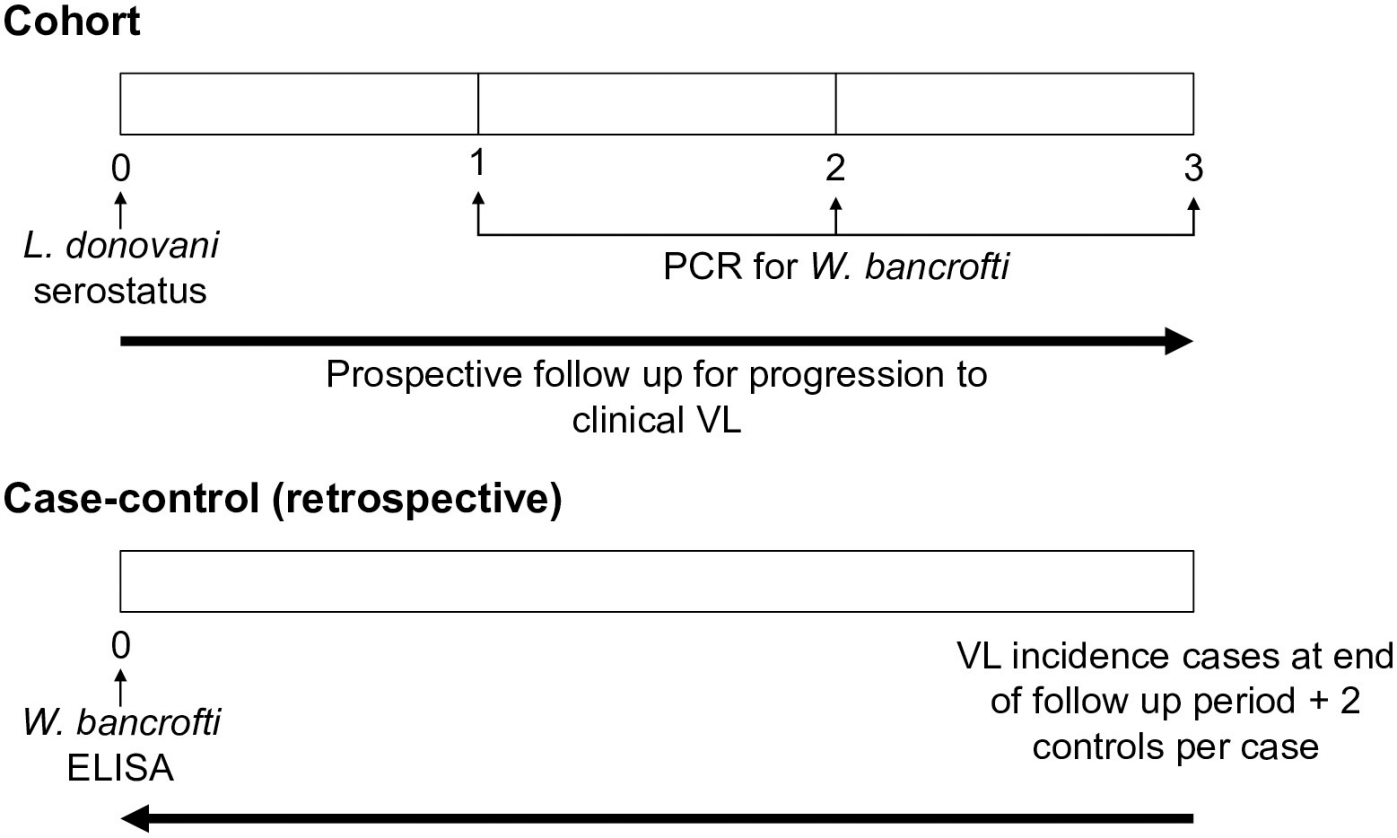
Illustration of cohort and case-control study designs.

We used both cohort and case-control study designs within the HDSS population to assess the prevalence of filarial infection in blood samples and examine the association between filarial infection and the progression to clinical VL (Figs 1 and 2).

A subset of 21,121 persons living in 27 villages highly endemic for VL in the Muzaffarpur TMRC HDSS area were enrolled in *L*. *donovani* serosurveys in 2009/10 [11]. From this, a cohort of 476 *Leishmania*-seropositive asymptomatic participants and 1,130 age- and village- group matched *Leishmania* seronegative healthy individuals were identified to be followed monthly for a minimum of three years to monitor the progression to clinical VL (previously described in [10]). To achieve sufficient numbers of seropositive participants, the cohort was recruited in stages–initially from 11 villages (approximately 13,200 individuals) then extended to 16 more villages (approximately 7,900 individuals)–after interim analysis showed the initial target sample size could not be reached in the first study area due to a decline in VL incidence [10]. All 27 villages were not appreciably different in their social or geographical characteristics or in VL disease incidence.

All participants, including those who were not included in the cohort (n = 10,515), continued to be monitored annually as part of the HDSS demographic surveys and followed-up monthly for new VL incidence. The retrospective case-control study design incorporated new VL cases (not otherwise identified as part of the cohort) along with two randomly selected controls (no record of progression to clinical disease in the same period) per case.

### Cohort study

In addition to monitoring for VL incidence in the cohort of 1,606 *Leishmania* seropositive or seronegative individuals, venous blood samples were collected annually for three years. Annual re-visits commenced between December 2010 and June 2011 in the first study area and continued for three years with the final sample collected in 2013–14. In the second study area, only two annual revisits occurred: visit 1 in 2012 and visit 2 in 2013–14 to complete the study in both the areas together. We used these diurnally collected blood samples for the retrospective detection of *Wuchereria bancrofti* microfilariae by real time PCR assay.

### Retrospective case-control study

A retrospective case-control study was undertaken by identifying all individuals who had been followed annually in the Muzaffarpur TMRC HDSS area and had been diagnosed with VL between 2010 and 2020, and two randomly selected controls (no record of progression to clinical disease in the same period) per case. Individuals already included in the cohort study described earlier, and those for whom a diurnal filter paper capillary blood sample from the start of the follow up period was not available were excluded. A total of 66 individuals who progressed to VL disease were identified in the Muzaffarpur TMRC HDSS follow-up area between 2010 and 2020, and we consequently selected 132 controls from the same population. Stored filter papers were only able to be retrieved for 36 VL cases and 71 controls (Fig 1). Insufficient blood samples were available to conduct a PCR assay to detect microfilaria; instead samples were tested for the presence of antibodies against the recombinant *W*. *bancrofti*-specific Wb123 antigen using an IgG4 enzyme linked immunosorbent assay (ELISA). VL clinical status was masked during the filaria testing and data entry.

### *Leishmania* serological status and progression to clinical visceral leishmaniasis

The *Leishmania* serological status of participants had been previously determined by detection of antibodies against *Leishmania* using direct agglutination test (DAT) and rK39-ELISA in samples collected at a first (2009–2011) and subsequent survey (after 6 to 12 months, depending on study area) [10]. Briefly, a participant was classified as a *Leishmania* seroconvertor (hereafter called seropositive) if they were serologically negative in first survey (DAT titre <1:1600 and rK39 ELISA OD< 14 percentage points (PP)), and positive with either test in the second survey (DAT titre ≥1:1600 or ELISA OD>14 PP with an increase of at least 2 titres in DAT and 3 PP in ELISA from the first serosurvey). A seronegative participant was rK39-ELISA and DAT-negative on both survey rounds.

Progression to clinical VL was confirmed parasitologically by splenic smear in participants who were clinically suspected during monthly follow-up (more than 2 weeks fever and rK39 RDT positive) [10].

### Detection of filarial infection

#### Detection of *Wuchereria bancrofti* DNA in peripheral blood by real time PCR assay

In order to determine the presence of *W*. *bancrofti* microfilariae in human blood, we conducted real-time PCR experiments using target sequences for the "long DNA repeat" of *W*. *bancrofti* (LDR; GenBank accession no. AY297458) [35]. The Primer Express software (Applied Biosystems, Foster City, CA) was used to construct the primers (LDR1, LDR2) and TaqMan probe (Table 1) for the LDR region sequence [36]. These primers and probe were synthesized commercially by IDT (Integrated DNA Technologies) (Coralville, Iowa, United States). This LDR probe was labelled with the FAM (6-carboxyfluorescein) reporter dye at the 5′ end and having two separate quenchers, a ZEN Internal Quencher between the ninth and tenth nucleotide bases of the oligonucleotide sequence and an Iowa Black FQ (3IABkFQ) at the 3’ end. In a total volume of 10 μl, real-time PCR experiments were carried out with 5 μl of TaqMan universal master mix from Applied Biosystems, 450 nmol/L of each primer, and 125 nmol/L of the probe and 4 μl of template DNA. The cycling settings for the testing procedure included an initial 2-minute incubation phase at 50°C for UNG activity, 10-minutes at 95°C for UNG deactivation and Taq polymerase activation, and 45 cycles of 15-second denaturation at 95°C, followed by 1-minute annealing and extension at 60°C. Thermal cycling and data analysis were done with an ABI 7500 instrument using SDS software (Applied Biosystems).

**Table 1:** Primer and probe "long DNA repeat (LDR)" target sequences for *W*. *bancrofti*.

Target group	Primer/ Probe	DNA sequence (5’→ 3’)
*W*. *bancrofti*	LDR-F	ATTTTGATCATCTGGGAACGTTAATA
	LDR- R	CGACTGTCTAATCCATTCAGAGTGA
	LDR- Probe	56-FAM/ATCTGCCCA/ZEN/TAGAAATAACTACGGTGGATCTCTG/3IABkFQ

The presence or absence of *W*. *bancrofti* DNA in each isolated sample was evaluated using the appropriate singleplex detection assay. The estimated Ct-value for each sample was determined and Ct-values greater than 40 were interpreted as negative whereby no parasite DNA was detectable in any of the samples.

#### *Wuchereria bancrofti*-specific antigen Wb123-based IgG4 ELISA

Detection of *W*. *bancrofti* specific antigens expressed by post-parasitic third-stage larvae have been demonstrated to be an early and specific marker of infection as they develop months to a year before appearance of blood microfilariae [37–39]. The Filaria Detect IgG4 ELISA kit (InBios, Seattle, WA), a direct enzyme immunoassay, was used to detect IgG4 antibodies against the recombinant Wb123 antigen in the preserved filter paper capillary blood samples. The experiment was carried out in accordance with the manufacturer’s standard operating procedures, with a few minor adjustments, as previously mentioned [38]. In summary, blood samples including positive and negative controls were diluted 1:50 in sample buffer given by the kit and stored at 4°C overnight. After adding samples and controls to plate wells, the plates were sealed and incubated at 37°C for 30 minutes. After incubation, the plates were washed using an automated plate washer and the wash buffer that came with the kit, incubated once more with mouse anti-human IgG4 conjugated with horseradish peroxidase for an additional 30 minutes, and then washed once more. After adding 100 μL of tetramethylbenzidine substrate to each well and incubating the plates at room temperature in the dark for 15 minutes, the reaction was stopped with the stop solution provided in the kit and the incubation was terminated after 1 minute. The microplate reader was set at 450 nm to read the plates. An optical density cut off of ≥2.037 was used. In the LF setting as found in India, it was difficult to identify 100 people with proven, filarial-positive disease as recommended by kit instructions to determine the cut off. Instead, the cut off for positive and negative results was derived from the mean optical density value obtained from all the kit Filaria Positive Controls used; this approach assured the avoidance of any false positivity with any potentially cross reactive diseases that could arise from the low positive control. We preferred a high antibody titre for filarial infection in this study.

### Data analysis

Data was double-entered by two independent data entry operators in a MS Access database and stored in a secure server in Banaras Hindu University. Participants were identified within MS Access by a unique ID which was used to match the results of laboratory analysis to demographic and clinical information. All data cleaning and analysis was completed using Stata BE 17.0 (StataCorp, USA).

Characteristics of participants included in the cohort and the case-control studies were analysed descriptively. Differences in proportions were assessed using a Pearson’s chi-squared test.

The risk of progression to VL as function of *W*. *bancrofti* infection status in the cohort study was assessed by calculating the risk ratios and 95% confidence intervals (95% CI). Although the cohort study included *Leishmania* seropositive and seronegative individuals who originally were frequency matched on age group and locality for an early study [10], it was not necessary to account for this matching in our analysis where the outcome is VL disease as a function of *W*. *bancrofti* infection, not *Leishmania* infection. For the case-control study we assessed the association between exposure to prior filarial infection and progression to clinical VL disease by calculating odds ratios and 95% CI. Chi-squared tests were applied to determine the significance of these associations. Statistical tests for associations and differences were determined to be significant when p<0.05.

Progression to VL was considered only when a diagnosis date was available for a participant. Persons who developed VL before their inclusion in the TMRC-HDSS cohort (including those with a relapse event recorded during the follow up period) were excluded from the main analysis. The filarial infection was considered as a risk factor for VL progression when demonstrated in a sample collected prior to date of diagnosis of clinical VL. Participants who did not have a sample collected at any time point or who had at least one sample collected during follow up, but did not have an LF result recorded in any year were excluded from the analysis.

## Results

### Cohort study

#### Cohort description

Of the 1606 individuals followed as part of the cohort study (476 asymptomatic participants seropositive for *Leishmania* and 1,130 frequency matched (age and locality) seronegative participants), at least one sample was available for LF testing from 1604 participants. Molecular testing for filarial infection was performed on samples from 1129 non-infected and 431 asymptomatic seroconverters. There were 44 participants with at least one sample collected who were not tested for LF in any year due to the small sample volume available; the majority of these were *Leishmania* seropositive participants. In total, there were 1560 cohort participants for whom at least 1 blood sample was analysed for *W*. *bancrofti* infection by PCR. Overall, there were 61% females in the LF-tested cohort; this proportion was the same between participants who were seronegative and asymptomatic *Leishmania* seropositive. The median age of the LF-tested seroconverters was 26 years as compared to 24 years for non-infected participants.

#### *Wuchereria bancrofti* infection

Overall, the prevalence of *W*. *bancrofti* infection (determined by PCR) was 11.5% over a three year follow up period (n = 179/1560 with at least one positive result). *W*. *bancrofti* PCR-positive participants were slightly older (median: 30 years, range: 3–80) than LF negative participants (median: 24 years, range: 2–88), and there was no difference in the proportion of *W*. *bancrofti* PCR-positive results between males and females.

The prevalence of *W*. *bancrofti* infection was not different between *Leishmania* seropositive (n = 52/431, 12.1%) and seronegative (n = 127/1129, 11.2%) individuals in the cohort study (p = 0.651).

#### Progression to clinical VL

Of the 1560 cohort participants for whom at least one blood sample was analysed for LF infection by PCR, 20 participants progressed to VL between 2010 and 2019 (Table 2; there were 10 participants who progressed to VL in each of the *Leishmania* seropositive and seronegative sub-cohorts). A significantly higher proportion of individuals who progressed to clinical VL were observed among those who were *Leishmania* seropositive compared with seronegative participants (Table 2; p = 0.024). There were 15 participants diagnosed with clinical VL within the LF sample collection period, and an additional 5 participants who were diagnosed between 2015 and 2019 as part of the routine HDSS follow-up activities. A higher proportion of males (60.0%) were observed among those participants who progressed to clinical VL in the follow-up period, compared to those who did not have clinical disease (38.7%; p = 0.052).

**Table 2:** Proportion of individuals who progressed to clinical VL in follow up period, by *Leishmania* serostatus at baseline (n = 1,560*).

*Leishmania* serostatus at baseline	Total	Progressed to VL	
		n	%
Seropositive	431	10	2.3%
Seronegative	1,129	10	0.9%

*number of cohort participants with at least one blood sample analysed for *W*. *bancrofti* infection by PCR, included in final analysis.

#### Risk of progression to clinical VL and *Wuchereria bancrofti* infection

The risk of progression to clinical VL was higher (RR: 2.57, 95% CI: 0.95–6.99) among filaria-infected individuals (determined by PCR) although this did not reach statistical significance (p = 0.056, Table 3). When accounting for *Leishmania* serostatus using adjusted (Mantel-Haenszel (M-H) combined) risk ratios, we found that participant *Leishmania* serostatus at the start of the follow up period neither modified nor confounded the association between filaria status and risk to progression (adj RR: 2.53, 95%CI: 0.93–6.86, p = 0.656).

**Table 3:** Analysis of risk of progression to clinical VL in follow up period and exposure to prior *Wuchereria bancrofti* (LF) infection (n = 1,560*).

LF PCR positive			LF PCR negative			RR	95% CI
Total	Progressed to VL	% at risk	Total	Progressed to VL	% at risk		
179	5	2.8%	1,381	15	1.1%	2.57	0.95–6.99

*number of cohort participants with at least one blood sample analysed for *W*. *bancrofti* infection by PCR, included in final analysis.

### Retrospective case-control study

#### Description of cases and controls

There were 36 VL clinical cases and 71 controls (no progression to VL recorded in follow-up period) included in the analysis. The median age of VL cases at recruitment (10 years, 95% CI: 5–15) was not significantly different from the control group (14 years, 95% CI: 10–20.3). There was no difference in the proportion of females in either group (53%).

#### Prevalence of *Wuchereria bancrofti* infection

Overall, the prevalence of IgG4 antibodies against the recombinant Wb123 antigen in the preserved blood plasma samples of the case-control study population at baseline was 24.3.% (n = 26/107). The prevalence of filarial infection did not significantly differ between males (20.0%, n = 11/55) or females (30.1%, n = 15/49) (p = 0.212; sex designation missing for 3 participants).

#### Odds of prior filarial infection in participants who progressed to clinical VL

Among individuals included in the retrospective case control study, the odds of having a prior filarial infection were significantly higher in participants who progressed to clinical VL (OR: 2.52, 95% CI: 1.02–6.25, p = 0.046, Table 4).

**Table 4:** Analysis of odds of prior *Wuchereria bancrofti* (LF) infection and progression to clinical VL in follow up period (n = 107).

Clinical VL			Controls			OR	95% CI
Total	LF ELISA +tve	% exposed	Total	LF ELISA +tve	% exposed		
36	13	36.1%	71	13	18.3%	2.52	1.02–6.25

## Discussion

With this study we have demonstrated an association between prior infection with *W*. *bancrofti* and the subsequent progression to clinical VL disease in the Muzaffarpur TMRC-HDSS area in Bihar state, India. Over a three year follow up period, the risk of progression to clinical VL was higher (RR: 2.57, 95% CI: 0.95–6.99) among *W*. *bancrofti* -infected individuals (determined by PCR) although this did not reach statistical significance (p = 0.056). When we sought to verify this finding using a retrospective case control study, the odds of having a prior *W*. *bancrofti* infection was significantly higher in participants who progressed to clinical VL (OR: 2.52, 95% CI: 1.02–6.25, p = 0.046).

This is the first study to our knowledge which has investigated the relationship between infection of two NTDs targeted for elimination. We were able to investigate our hypothesis using two different study designs and support our previous findings from a demographic survey in the Muzaffarpur TMRC-HDSS area in 2013 which identified that the prevalence of self-reported signs of LF was significantly higher (10.4%) among those who had suffered from VL compared the overall HDSS population (5.1%). In all instances our sample was drawn from a large well-established HDSS population in a VL/LF co-endemic area. As the Muzaffarpur TMRC-HDSS incorporates intensive longitudinal follow up of participants to identify progression to clinical VL, we assume that this minimised the number of clinical VL cases missed.

The impact of helminths on other co-infections and vaccinations remains inconclusive, but many point to parasitic worms negatively impacting other infections and vaccination [40–44]. Studies on helminth-*Leishmania* co-infection are few, but in general indicate that worms can facilitate leishmanial infection [40,45]. Evidence for a sequential relationship between LF or VL infection and development of clinical disease has previously not been shown, likewise the role of co-infection in disease pathology is not well understood. A study from Mali addressing the prevalence of filaria (by *W*. *bancrofti* immunochromatographic test (ICT)) and *Leishmania* infection (*Leishmania* skin test (LST) reaction to *L*. *major* infection) found slightly higher prevalence of LST positive reactions in ICT positive individuals [46]. While this study was too small to draw definitive conclusions, it, in line with the data presented here, suggest that further investigation on the role filarial co-infections have on concomitant diseases is warranted.

The influence of the immune response to prior *Leishmania* infection on subsequent susceptibility to filarial infection is also not well defined. We found that the prevalence of baseline filarial infection did not differ between *Leishmania* seronegative (11.2%) and seropositive (12.1%) individuals included in the cohort study. The influence of *Leishmania* seropositivity may be short lived – more than 80% asymptomatically infected individuals in 26 high-endemic villages in India and Nepal followed as part of the Kalanet study were found to have turned seronegative again within a year [6]. Likewise, we found the risk of progression to clinical VL in LF-infected individuals was not affected by *L*. *donovani* serostatus measured at recruitment, suggesting the effect of progression could more likely be attributed to *W*. *bancrofti* infection. Large cohort studies in Nepal and India have demonstrated a strong association between serostatus (high *Leishmania* antibody titres) and development of clinical disease, and a recent seroconversion was also associated with a risk of developing symptomatic VL [10,11]. We observed an influence of male sex in the risk of progression to clinical VL in the cohort component of this study, consistent with a previous study evaluating large population surveillance project datasets, including from the HDSS population, which suggested that biological differences between men and women may play a critical role in the pathogenesis of VL [47].

There are some limitations which must be considered when interpreting the results of this study, which drew on the existing design of a large HDSS cohort, and was conducted retrospectively. We did not specifically measure filarial and *Leishmania* co-infection in the samples taken at the same time points, thus reducing the certainty of temporal associations when attributing causality between co-infection and the influence of progression to either disease. Furthermore, our retrospective case-control analysis only examined filarial infection at a single sample and could not account for additional instances of filarial infection prior to the development of clinical VL other than that evident at the initial point of recruitment (baseline), and our cohort study included a longer follow of period for assessing progression to clinical VL than for serological measurement of filarial infection, meaning some interim filarial infections between the conclusion of sample collection and development of clinical disease may have been missed. It will be important in future research to also incorporate an examination of the temporal influence of *Leishmania* infection on immunomodulation and the development of LF pathology, previously suggested in a mouse model of *Brugia malayi* infection where immunization with *L*. *donovani* molecules increased filarial parasite burden [48].

We used molecular and immunological methods to detect microfilariae in retrospective samples collected in this study. While we had insufficient sample to assess filarial infection using the same diagnostic test for each study component, we are still able to make relative comparisons of infection rates in each sub-study. The diurnal collection of blood samples in both the cohort and case-control study populations may have limited the detection of the microfilariae that cause lymphatic filariasis which peak between 23:00 h and 01:00 h and decline to the lowest levels between 12:00 h and 15:00 h [49], thus underestimating the number of LF-infected individuals. Although we acknowledge the suboptimal sensitivity of the methodology used, this should not affect specificity; we can therefore assume the association identified through this study to be a valid one.

Restricting our definition of LF infection by the detection of microfilariae excludes asymptomatic or subclinical infections of LF which have been described in people with filarial antigenemia (a surrogate for viable adult worms) in the absence of microfilariae [19,50]. Additionally, the identification of LF-related clinical events in conjunction with microfilariae and filarial antigenemia would have provided further information to allow the differentiation of asymptomatic infections among the study populations. This would have been important to extend our understanding of the immunomodulatory role of LF infection on the risk of progression to VL based on clinical status of LF: asymptomatic LF patients develop a regulatory type 2 immune response while LF patients with pathology develop pro-inflammatory type 1 immune responses [18,19] and the immune response among microfilariae positive and microfilariae negative LF patients without pathology differs [51].

Finally, other chronic worm infections, such as soil-transmitted helminths (STH), are known to be drivers of regulatory responses needed to limit infection-induced tissue pathology [15,16], and co-infection with intestinal helminths has also been found to influence the clinical outcome and immune response of patients with *Leishmania* infection manifesting as CL [22]. The role of STH infection in impairing protective immune responses against VL, and any confounding or effect modification of the association between LF infection and the progression to VL, was not investigated in our current study. We note a scoping study by some of the co-authors of the current study which compared gut flora in VL cases (N = 23) and endemic controls (EC; N = 23) from Muzaffarpur in Bihar State [52] found a high prevalence (45%) of carried intestinal helminths, with no overall difference between VL cases or EC. Although STH were among the intestinal helminths identified, the authors indicated that their scoping study was underpowered to determine any association of STH with VL.

India carries the vast majority of both VL and LF cases in the South-East Asia region. Our findings highlight the need for an integrated approach for Neglected Tropical Diseases in co-endemic areas. Reducing filarial disease exposure through mass drug administration (MDA) may be an important way to reduce the transmissibility of filarial and risk of progression of clinical VL infection, especially in areas with overlapping disease distribution. The benefits of considering the integration of disease programs for *Leishmania* and lymphatic filariasis extend beyond the potential relationship of sequential or co-infection with disease burden. Integrated programs for control and elimination of NTDs such as leishmaniasis and lymphatic filariasis in areas of co-endemicity benefit from overlapping vector control activities, health system networks and surveillance infrastructure.

This study strongly suggests there is an increased risk of progression to clinical VL in individuals with a history of infection with *W*. *bancrofti*. A prospective cohort study on asymptomatic *Leishmania* infected individuals is underway to assess whether *W*. *bancrofti* co-infected individuals have a higher risk of progressing to VL over a three-year follow-up period than non-co-infected individuals in the Muzaffarpur HDSS area, and to study the risk factors and immunomodulatory factors associated with this co-infection.
